# Health Personality and Well-Being: An Overview

**DOI:** 10.1093/geroni/igaa057.2163

**Published:** 2020-12-16

**Authors:** Peter Martin, Joseph Kim, Rotem Arieli, Nicholas Cone

**Affiliations:** Iowa State University, Ames, Iowa, United States

## Abstract

It is well established that there are interindividual differences in many areas of well-being. Based on previous segmentation research and work connecting personality traits to health outcomes, we developed the health personality segmentation model. The health personality segmentation model takes a person-centered approach to provide optimal health care based on segmenting individuals through health personality factors. The presentation provides an overview of health personality, defined as a set of individual dispositions that are directly related to health. We introduce the Health Personality Assessment based on our health personality segmentation model, and we link health personality with resilience, activation, as well as with physical and emotional well-being. Future research should evaluate the usefulness of this assessment in applied healthcare settings.

